# Response to “The promise and pitfalls: interpreting dual β-lactam success in *Mycobacterium abscessus* with caution”

**DOI:** 10.1128/asmcr.00170-25

**Published:** 2025-12-09

**Authors:** Khalid M. Dousa, Robert A. Bonomo

**Affiliations:** 1 Louis Stokes Cleveland VA Medical Center, Case Western Reserve University 2546, Cleveland, Ohio, USA; 2 Department of Medicine, Case Western Reserve University School of Medicine 12304, Cleveland, Ohio, USA; 3 CWRU-Cleveland VAMC Center for Antimicrobial Resistance and Epidemiology (Case VA CARES) 2546, Cleveland, Ohio, USA; 4 Department of Pharmacology, Case Western Reserve University School of Medicine 12304, Cleveland, Ohio, USA; 5 Department of Biochemistry, Case Western Reserve University School of Medicine 12304, Cleveland, Ohio, USA; 6 Department of Proteomics and Bioinformatics, Case Western Reserve University School of Medicine 12304, Cleveland, Ohio, USA; 7 GRECC, Louis Stokes Cleveland Department of Veterans Affairs Medical Center 20083, Cleveland, Ohio, USA; Pattern Bioscience, Austin, Texas, USA

**Keywords:** *Mycobacterium abscessus*, β-lactam, β-lactam lactamase inhibitors, clinical trial

## Abstract

We read with interest the recent commentary (C. Mejia-Chew, ASM Case Rep 1:e00084-25, 2025, https://doi.org/10.1128/asmcr.00084-25) on our case report (M. Shimamura, B. Becken, J. Tansmore, M. Cristinziano, et al., ASM Case Rep 1:e00087-24, 2025, https://doi.org/10.1128/asmcr.00087-24). β-lactam therapy represents a mechanistically rational and clinically promising strategy for *Mycobacterium abscessus*, a pathogen with limited therapeutic options. Reported cases of β-lactam combinations illustrate how *in vitro* data, translational insights, and multidisciplinary clinical decision-making can inform salvage therapy in the absence of robust animal preclinical or clinical trial data. While challenges remain—including lack of standardized synergy testing, limited animal models, and complex polytherapy—these experiences underscore the importance of transparent reporting and provide the platform for rigorously evaluating β-lactam regimens.

## COMMENTARY

We appreciate the recent engagement with our case report (1) and share the author’s central aims: to reduce therapeutic nihilism in *Mycobacterium abscessus* (*Mab*) while supporting rigorous evidence. Where we differ is in (i) the description of clinical observations and (ii) the suggestion that early clinical signals warrant substantial skepticism despite transparent reporting.

Firstly, let us review how the current recommendations from the Infectious Diseases Society of America (IDSA) and the American Thoracic Society (ATS) for nontuberculous mycobacteria have evolved. Surprisingly, for *Mab*, a randomized clinical trial that validates the majority of the multidrug regimens currently used in practice has never been performed (2). Guideline recommendations—macrolides, amikacin, and others—rest largely on *in vitro* data, observational series, and expert consensus (2). *Mab* therapeutics have advanced through careful laboratory rationale, multidisciplinary clinical debate, and transparent case documentation. Evaluating current novel therapies (i.e., double β-lactams) raises questions regarding the best way to incorporate new findings into clinical care.

Secondly, publication bias exists—not only here, but in many fields. Case reports in *Mab* are rarely casual anecdotes; they are the “tip of an iceberg” of interdisciplinary work: pharmacology reviews, susceptibility and synergy testing where available, repeated regimen adjustments, longitudinal safety assessments, and—quite often—hard decisions in the face of toxicity or failure of standard options. Rather than presume these efforts inflate efficacy, the more productive stance is to expand the denominator through prospective registries with pre-specified fields (e.g., subspecies, *erm*/inducible macrolide resistance status, surgical interventions, bacteriophage use, dual β-lactam (DBL) combination and dosing, therapeutic drug monitoring where applicable, microbiologic endpoints, radiographic and patient-reported outcomes, and safety). Most of us would be eager to contribute data to such a framework.

Thirdly, the concerns about standardized synergy testing and site-of-infection pharmacodynamics are well-taken—and they apply to virtually every *Mab* regimen. The absence of a CLSI-standardized DBL synergy method does not mean we should ignore concordant laboratory signals. A pragmatic path is to (i) establish a single-agent minimal inhibitory concentration using standardized, CLSI-aligned methods; (ii) when feasible, perform reproducible synergy screens (e.g., checkerboard or time-kill with internal controls) in reference labs familiar with *Mab*; (iii) interpret DBL choices through known biochemistry (Bla_Mab_ hydrolysis kinetics, D,D- vs L,D-transpeptidase targeting, β-lactamase inhibitor spectra); and (iv) pair that with plausible PK/PD exposures at the pulmonary site (noting that some β-lactams and diazabicyclooctane inhibitors achieve clinically relevant free-drug concentrations in epithelial lining fluid). None of this substitutes for clinical trials. It is the best synthesis available in a disease where animal models remain difficult to generalize and where patients cannot wait for perfect systems.

Fourth, on attribution in multi-modal care warrants careful consideration. We agree that isolating the effect size of any single intervention in *Mab* is challenging when courses include sequential or concurrent drugs, surgery, and even bacteriophage therapy. But this is a reason to design analyses that model multi-component care (e.g., hierarchical, time-varying covariate approaches in registries), not a reason to discount signals altogether. Methodological tools exist—Bayesian borrowing, causal inference with marginal structural models, and N-of-1 designs embedded in platforms—that can make better use of the real-world complexity rather than treat it as a disqualifier (3).

Fifth, history matters. Infectious diseases have repeatedly moved from toxic “standards” toward safer, mechanism-informed combinations after case-based and laboratory signals accumulated. The evolution in *Enterococcus faecalis* endocarditis from aminoglycoside-containing regimens to ampicillin-ceftriaxone—motivated by target redundancy and supported by translational rationale—illustrates how careful mechanistic thinking, combined with clinical observation, can re-route practice even before large randomized controlled trials (RCTs) are feasible (4–6). DBL strategies for *Mab* are conceptually analogous: complementary inhibition of transpeptidase pathways and protection from Bla_Mab_ hydrolysis can plausibly convert partial activity into a clinically meaningful effect, at least for some patients and subspecies (7, 8).

Sixth, we fully endorse the call for rigorous trials and welcome the FORMaT platform as a critical step forward (3). We suggest that DBL arms be prospectively incorporated in ways that reflect the laboratory rationale and emerging clinical practice: (i) pre-specify a small set of DBL pairs with the strongest mechanistic support; (ii) stratify by subspecies and macrolide resistance mechanisms; (iii) use adaptive randomization with early futility and safety stops; and (iv) include microbiologic, functional, and patient-reported outcomes that matter in a disease with prolonged treatment horizons. Parallel registries can capture broader safety and generalizability, especially in populations that trials will under-represent.

Finally, a more constructive path forward is to invite comprehensive reporting, enable shared methods for synergy and PK/PD interpretation, and accelerate entry of promising DBL combinations into adaptive platforms—so that we can, together, confirm, refine, or retire them on the basis of data.

In sum, we are aligned in our goal: to achieve safer, more effective, and evidence-based therapy for *Mab*. This is how the evidence base for *Mab* has historically evolved, and how it will, we believe, ultimately lead to improved outcomes for patients (9).
